# Quantitative trait locus analysis for spikelet shape-related traits in wild wheat progenitor *Aegilops tauschii*: Implications for intraspecific diversification and subspecies differentiation

**DOI:** 10.1371/journal.pone.0173210

**Published:** 2017-03-06

**Authors:** Ryo Nishijima, Yuki Okamoto, Hitoshi Hatano, Shigeo Takumi

**Affiliations:** Laboratory of Plant Genetics, Graduate School of Agricultural Science, Kobe University, Nada, Kobe, Japan; Institute of Genetics and Developmental Biology Chinese Academy of Sciences, CHINA

## Abstract

Wild diploid wheat *Aegilops tauschii*, the D-genome progenitor of common wheat, carries large genetic variation in spikelet and grain morphology. Two differentiated subspecies of *Ae*. *tauschii*, subspecies *tauschii* and *strangulata*, have been traditionally defined based on differences in spikelet morphology. Here, we first assessed six spikelet shape-related traits among 199 *Ae*. *tauschii* accessions, and found that the accessions belonging to TauL1major lineage produced significantly longer spikes, higher spikelet density, and shorter, narrower spikelets than another major lineage, TauL2, in which the *strangulata* accessions are included. Next, we performed quantitative trait locus (QTL) analysis of the spikelet and grain shape using three mapping populations derived from interlineage crosses between TauL1 and TauL2 to identify the genetic loci for the morphological variations of the spikelet and grain shape in *Ae*. *tauschii*. Three major QTL regions for the examined traits were detected on chromosomes 3D, 4D and 7D. The 3D and 4D QTL regions for several spikelet shape-related traits were conserved in the three mapping populations, which indicated that the 3D and 4D QTLs contribute to divergence of the two major lineages. The 7D QTLs were found only in a mapping population from a cross of the two subspecies, suggesting that these 7D QTLs may be closely related to subspecies differentiation in *Ae*. *tauschii*. Thus, QTL analysis for spikelet and grain morphology may provide useful information to elucidate the evolutionary processes of intraspecific differentiation.

## Introduction

*Aegilops tauschii* Coss. (formerly called *Ae*. *squarrosa* L.), the D-genome progenitor of common wheat, is a wild diploid wheat relative with a wide distribution range from northern Syria and southeastern Turkey to western China [1,2]. The genome of *Aegilops tauschii* was brought into common wheat through interspecific crossing to tetraploid emmer wheat and subsequent amphidiploidization about 8,000 years ago [3]. This evolutionary process can be artificially reproduced by generation of synthetic wheat hexaploids, obtained through interspecific triploid hybrids from crosses between cultivated tetraploid wheat and *Ae*. *tauschii* [4,5]. Therefore, *Ae*. *tauschii* is an useful genetic resource for wheat breeding to transmit phenotypic variation to common wheat through synthetic wheat hexaploids [6–9].

*Ae*. *tauschii* populations provide large natural variation in spikelet and floral morphological traits as well as flowering time [2,10–12]. Two subspecies, *Ae*. *tauschii* Coss. subspecies *tauschii* and *Ae*. *tauschii* Coss. subspecies *strangulata* (Eig) Tzvel., have been recognized in *Ae*. *tauschii* based on spikelet morphology [13,14], whereas the two typical forms of subspecies *tauschii* and *strangulata* are connected by a continuous range of intermediate forms [15]. Some reports indicated difficulty in distinguishing the two subspecies, suggesting a high level of gene flow between the subspecies [16–18]. On the other hand, subspecies divergence was observed using the sensu-stricto criteria for subspecies *strangulata* in the classification [10,11]. For the sensu-stricto classification, the subspecies was defined based on classical reports [13,14]; accessions of subspecies *tauschii* have elongated cylindrical spikelets, whereas subspecies *strangulata* is characterized by quadrate spikelets. Moreover, natural variation in spikelet-related traits of *Ae*. *tauschii* shows significant longitudinal and latitudinal clines for spikelet size, with spikelets tending to be small in eastern and southern regions [10,11]. Overall, spikelet morphology is the key characteristic not only for differentiation of the two subspecies but also for intraspecific diversification in *Ae*. *tauschii*, although the genetic basis of spikelet shape divergence has not yet been analyzed.

Recent Bayesian population structure analyses with genome-wide marker genotyping showed that *Ae*. *tauschii* could be divided into two major genealogical lineages, *tauschii* lineage 1 (TauL1) and TauL2, and a minor lineage, TauL3 [12,19–21]. The TauL1 accessions are distributed from western habitats in the Transcaucasian and northern Iran regions to eastern habitats such as Pakistan and Afghanistan, whereas TauL2 is restricted to western habitats and subspecies *strangulata* is involved only in TauL2 [19,22]. Thus, differentiation of subspecies *strangulata* is considered to have occurred in TauL2. Moreover, Bayesian structure analyses indicate that two sublineages have genetically diverged in each of the two lineages, TauL1 and TauL2 [21]. The eastward species expansion appears to be driven by the broad habitat range of TauL1, especially by one of the sublineages, Tau1b (TauL1b), and the TauL1b accessions exhibit peculiar phenotypes with early flowering time, high seed production ability, and salt stress tolerance during seedling growth [12,23]. Such morphological and physiological divergence might also underlie other traits that differ between TauL1 and TauL2 or among sublineages, because spikelet size tends to be small and density high in the eastern habitats [10,11]. Early-flowering accessions also have spread mainly in the eastern habitats [2,12], and genetic variation related to the early flowering phenotype have been partly studied [24–26]. However, no loci related to intraspecific divergence, especially for the *Ae*. *tauschii* morphology, have been identified.

Wheat spikelet morphology is related to grain shape and size, and spikelet shape-controlling genes pleiotropically affect grain shape [27,28]. Grain shape and size have been two of the main targets for wheat domestication and breeding [29,30], and thus understanding the genetic mechanisms controlling spikelet morphology is important for wheat breeding as well as intraspecific diversification in wheat relatives. The aims of the present study were (1) to clarify the relationships of the natural variation in spikelet and grain shape with the genetic lineages and (2) to elucidate the genetic loci controlling the morphological differences between the two subspecies and between the lineages in the wild D-genome progenitor of common wheat. In the present study, therefore, we conducted quantitative trait locus (QTL) analysis for spikelet shape-related traits using three mapping populations to identify the genetic loci for morphological variation in *Ae*. *tauschii*. Based on results of the QTL analyses, the evolutionary processes of lineage divergence and subspecies differentiation in *Ae*. *tauschii* were reevaluated.

## Materials and methods

### Plant materials

Phenotypic data on spikelet morphology-related traits of the 199 accessions of *Ae*. *tauschii* were based on our previous reports [10,11]. The *Ae*. *tauschii* accessions were genealogically divided into two major intraspecific lineages, TauL1 and TauL2, and a minor lineage, TauL3, and TauL1 and TauL2 were each further divided into two sublineages; TauL1a and TauL1b, and TauL2a and TauL2b [12,21]. For comparison of the examined traits between the lineages and among the sublineages, data points on the TauL3 accessions and admixtures in TauL1 and TauL2 were omitted due to their limited numbers. The genealogical lineage and sublineage information of each *Ae*. *tauschii* accession was based on our previous report [12,21]. The phenotypic data and lineage information are represented in S1 Table.

**Fig 1:**
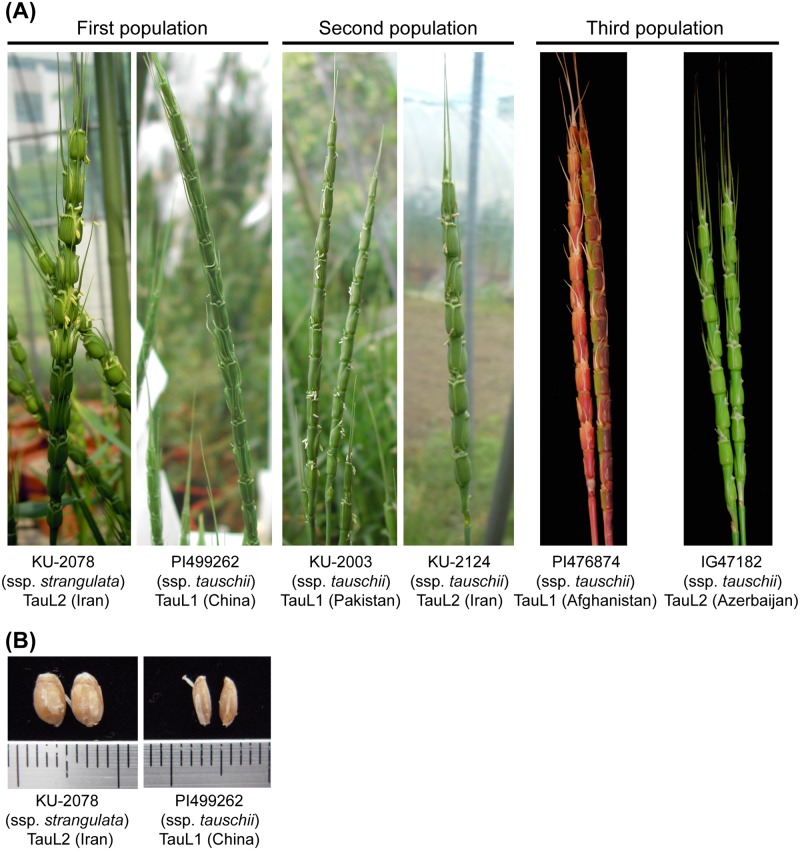
Photos of the parental accessions of the mapping populations. (A) Spike phenotype of each parental accession. (B) Grain shape of the two subspecies.

Three F_2_ mapping populations of *Ae*. *tauschii* were used: KU-2078/PI499262, KU-2003/KU-2124, and PI476874/IG47182. In the parental accessions of the mapping populations, KU-2003, PI476874, and PI499262 belong to TauL1, and KU-2124, IG47182, and KU-2078 belong to TauL2 (Fig 1). The parental cross-combinations of all three populations were selected from interlineage pairs TauL1 and TauL2. KU-2078 is classified in subspecies *strangulata*, and the other five accessions are in subspecies *tauschii*. Therefore, the first population (KU-2078/PI499262) resulted from an intersubspecies cross between subspecies *strangulata* and subspecies *tauschii*, and the second and third (KU-2003/KU-2124 and PI476874/IG47182) populations were derived from intrasubspecies crosses of subspecies *tauschii*.

Plants were arranged randomly and grown in the experimental field of Kobe University. Seeds of the first F_2_ population (KU-2078/PI499262) were sown in November 2012, with a population size of 95. The second population (KU-2003/KU-2124) contained 116 F_2_ individuals and was grown in the 2011–2012 season [22]. The last population (PI476874/IG47182), with 104 F_2_ individuals, was grown in the 2008–2009 season [31].

### Evaluation of spike, spikelet and grain morphology

For 39 randomly selected accessions from the 199 *Ae*. *tauschii* accessions, four grain shape-related traits, grain length (GL), grain width (GW), grain height (GH), and length-to-width ratio of the grain (LWr), were measured. For each accession grown under field conditions at Kobe University in the 2009–2010 season, the grain shape-related traits of more than 20 seeds were measured, and the averages and standard deviations were calculated for each measurement. The phenotypic data for spikelet and grain morphology-related traits of the 199 accessions were statistically analyzed using RStudio ver. 0.99.902 [32] in R software ver. 3.3.1 [33] for Welch’s *t* test, Steel-Dwass test, and principal component analysis (PCA), and R package ‘ggplot2’ [34] for PCA ploting.

For the first and second mapping populations, KU-2078/PI499262 and KU-2003/KU-2124, eight spikelet-related traits, i.e. spike length (SL), number of spikelets per spike (NSp), number of immature spikelets per spike (NISp), spikelet density (SpD), spikelet length (SpL), spikelet width (SpW), empty glume length (EGL), empty glume width (EGW), and four grain-related traits of GL, GW, GH, and LWr were evaluated. For the last population (PI476874/IG47182), six spikelet-related parameters (SL, NSp, SpD, SpL, EGL, and EGW) were measured. The three tillers that headed earliest for each F_2_ individual were used to measure the morphological traits, and the averages and standard deviations were calculated for each. The phenotypic data were statistically analyzed using R software.

### Marker information and genotyping

To amplify PCR fragments of single sequence repeat (SSR) markers, total DNA was extracted from leaves of the parental *Ae*. *tauschii* accessions and F_2_ individuals. Information on SSR markers and the respective annealing temperatures was obtained from the National BioResource Project (NBRP) KOMUGI web site (http://www.shigen.nig.ac.jp/wheat/komugi/strains/aboutNbrpMarker.jsp) and the GrainGenes web site (http://wheat.pw.usda.gov/GG2/maps.shtml). For SSR genotyping, 2x Quick Taq HS DyeMix (TOYOBO, Osaka, Japan) was used as a master mix for the reactions under the following conditions: 40 cycles of 10 s at 94°C, 30 s at the annealing temperature for each SSR marker, and 30 s at 68°C. Information on the ‘kupg’ SSR markers on chromosome 7D is in our previous reports [35,36]. Six PCR-based landmark unique gene (PLUG) markers, TNAC, on chromosomes 3D and 7D were used additively according to a previous report [37]. Genotyping with 16 ‘ctg’ single nucleotide polymorphism (SNP) markers were conducted according to our previous report [31].

For a cleaved amplified polymorphic sequence (CAPS) marker, Bd50050 on chromosome 7D, PCR products amplified with the primer set 5’-CTGCTGCGCCATTCTATTC-3’ and 5’-TAGAATGCAAGGGTGGCAAT-3’, then digested by the 4-bp cutting restriction enzyme *Hha*I. For mapping of the leaf rust-resistance locus *Lr34* on 7DS [38], the primer set 5’-TGCGGCGATTCTATACTACT-3’ and 5’-CCGACATCAAGAACCTCC-3’ was used, and the PCR-amplified products were digested by the 4-bp cutting enzyme *Taq*I.

PCR products and their digests were resolved in 2% agarose or 13% nondenaturing polyacrylamide gels, stained with ethidium bromide, and visualized under UV light.

A phenotypic difference for hairy character on the flag leaf was observed between the parental accessions of the first mapping population, and the causal gene, tentatively named *hfl*, was used as a genetic marker for chromosome 3D.

### Linkage map construction and QTL analysis

The MAPMAKER/EXP version 3.0 package was used for construction of genetic maps with the genotyped markers [39]. The threshold for log-likelihood (LOD) scores was set at 3.0, and genetic distances were calculated with the Kosambi function [40]. QTL analyses were carried out by composite interval mapping with Windows QTL Cartographer version 2.5 software [41] using the forward and backward method. A LOD score threshold for each trait was determined by computing a 1000 permutation test. The percentage of phenotypic variation explained by a QTL for a given trait and any additive effects were also estimated.

## Results

### Intraspecific and intersubspecies variation in spikelet-related traits

**Fig 2:**
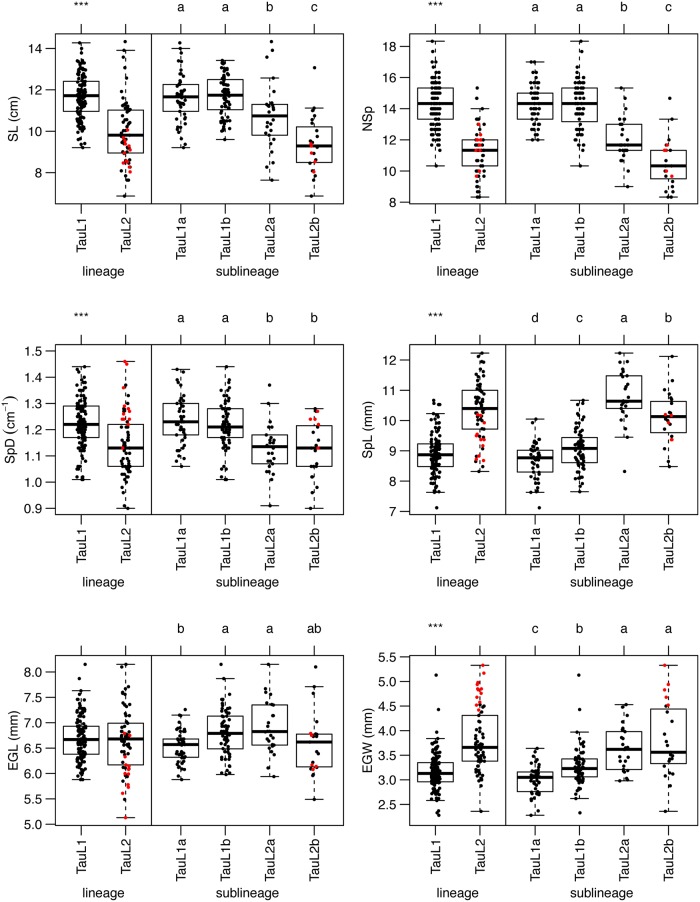
Box and dot plots for the six spikelet-related traits based on the lineages and sublineages. Welch’s *t*-test was conducted for statistical significance (***P < 0.001) of differences between the two lineages, TauL1 and TauL2. Sublineages sharing a common letter were not significantly different (Steel-Dwass test, *P* < 0.05). The tests were performed excluding TauL3 in the interlineage analyses and TauL1x and TauL2x in the intersublineage analyses because of the lack of statistical power. Red and black dots indicate the accessions of subspecies *strangulata* and *tauschii*, respectively.

Large divergence in spikelet shape-related traits has been observed between subspecies *tauschii* and *strangulata* [10,11]. Floral organ shape and culm-related traits also diverge between the subspecies [11]. The six spikelet shape-related traits, SL, NSp, SpD, SpL, EGL, and EGW, analyzed in the present study showed large natural variation in 199 accessions of *Ae*. *tauschii* [10,11]. Morphological variation was assessed based on lineage and sublineage groups for the six spikelet shape-related traits. Significant differences (Welch’s *t*-test, *P* < 0.001) were observed between TauL1 and TauL2 for five traits, SL, NSp, SpD, SpL, and EGW, but not for EGL (Fig 2). Multiple comparisons among the four sublineages (TauL1a, TauL1b, TauL2a, and TauL2b) showed significant differences (Steel-Dwass test, *P* < 0.05) in the six traits even within the same lineages. The SpL value differed significantly between all four sublineages. Although there was no significant difference in EGL between the lineages, the sublineages TauL1b and TauL2a showed significantly higher EGL values than TauL1a. Significant differences were found between TauL1 sublineages for EGW and between TauL2 sublineages for SL and NSp. No significant difference for SpD was observed within the two sublineages in each lineage.

**Fig 3:**
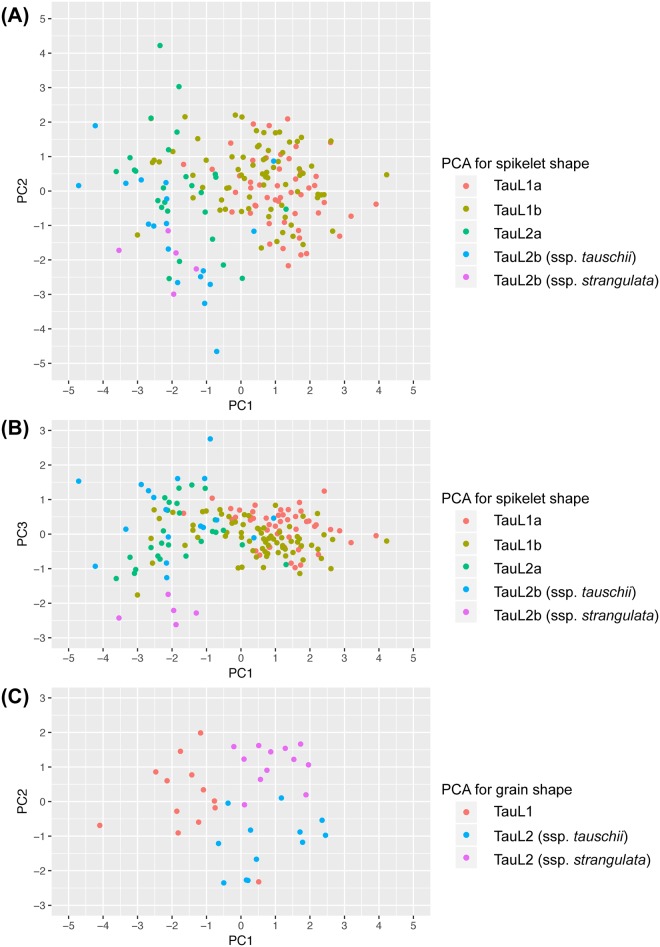
Graph of the first three and two axes from two principal component analyses based on the six spikelet shape-related (A, B) and four grain shape-related (C) traits.

To clarify the relationship between spikelet shape and the sublineages, PCA was conducted based on the correlation matrix among the *Ae*. *tauschii* accessions. The first principal component (PC1) explained 50.3% of the total variance, and the variation in PC1 values had major effects on NSp, SpD, SpL and EGW (Table 1). PC2 explained 28.0% of the total variance, and high eigenvector values for PC2 were found in SL, NSp and EGL. PC3 contributing 10% of the variance was mainly derived from eigenvectors of SpD and EGW. The plot of the 169 accessions using their PC1 and PC2 values showed that the TauL1 accessions had generally larger PC1 values than the TauL2 accessions (Fig 3A), indicating that TauL1 tended to have longer spike, more spikelets per spike, and smaller spikelet than TauL2, which was consistent with the previous report [12]. The PC2 values in TauL2 were more widely distributed than those in TauL1. Two sublineages of TauL2, TauL2a and TauL2b, were slightly differentiated, whereas the sublineage diversification was not clear in TauL1. Although the subspecies differentiation was not found in the graph of the first two axes from the PCA, subspecies *strangulata* formed a cluster in a graph using the PC1 and PC3 values (Fig 3B). The PC1-PC3 graph showed the differentiation of subspecies *strangulata* from subspecies *tauschii* in TauL1. These results indicated that these spikelet shape-related traits were related to the sublineage divergence and subspecies differentiation of *Ae*. *tauschii*.

**Table 1:** Eigenvectors for the first and second principal components for the six spikelet-related traits.

Trait	PC1	PC2	PC3
Spike length	0.24	0.67	−0.02
Number of spikelet per spike	0.46	0.43	−0.27
Spikelet density	0.46	−0.24	−0.46
Spikelet length	−0.51	0.18	0
Empty glume length	−0.29	0.52	0.04
Empty glume width	−0.42	0.01	−0.84

PC1, PC2, and PC3 explain 50.3, 28.0, and 10.0% of the total variance, respectively.

**Fig 4:**
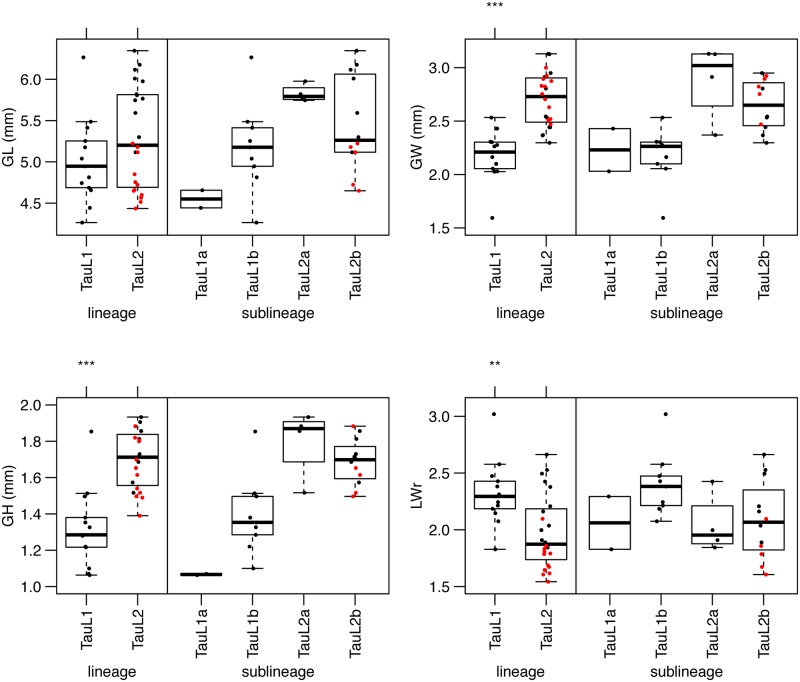
Box and dot plot comparison of grain shape between lineages and among sublineages of *Ae*. *tauschii*. Welch’s *t*-test was conducted for statistical significance (***P < 0.001) of differences between the two lineages, TauL1 and TauL2. Multiplex comparison between the four sublineages was excluded because of the lack of statistical power. Red and black dots indicate the accessions of subspecies *strangulata* and *tauschii*, respectively.

Four grain shape-related traits, GL, GW, GH and LWr, varied in the randomly selected 39 accessions of *Ae*. *tauschii*, and significant differences (Welch’s *t*-test) were observed between TauL1 and TauL2 for GW and GH (*P* < 0.001) and for LWr (*P* < 0.01) (Table 2 and S1 Table). Grain size tended to be larger and LWr was smaller in TauL2 than in TauL1. The two sublineages of each lineage showed similar values for the four traits, although the number of examined accessions of each sublineage was limited (Fig 4). Three of the four grain shape-related traits also showed significant divergences (Welch’s *t*-test, *P* < 0.001) between the two subspecies (Table 2). GL was longer in subspecies *tauschii* than in subspecies *strangulata*, whereas GW was wider in subspecies *strangulata* than in subspecies *tauschii*. LWr was significantly smaller in subspecies *strangulata* than in subspecies *tauschii*, indicating that grains of subspecies *strangulata* were more spherical than those in subspecies *tauschii*.

**Table 2:** Grain shape variation in lineages and subspecies of *Ae*. *tauschii*.

Grain traits	Lineage		
	TauL1 (n = 13)	TauL2 (n = 24)	TauL1 + TauL2
Grain length (mm)	5.01 ± 0.52	5.29 ± 0.61	5.20 ± 0.59
Grain width (mm)	2.18 ± 0.23	2.70 ± 0.25***	2.52 ± 0.35
Grain height (mm)	1.32 ± 0.22	1.70 ± 0.16***	1.57 ± 0.26
Length/width ratio	2.32 ± 0.28	1.98 ± 0.32**	2.10 ± 0.35
Grain traits	Subspecies		
	*tauschii* (n = 27)	*strangulata* (n = 12)	Total
Grain length (mm)	5.39 ± 0.59	4.77 ± 0.27***	5.2 ± 0.58
Grain width (mm)	2.39 ± 0.36	2.74 ± 0.18***	2.5 ± 0.35
Grain height (mm)	1.51 ± 0.29	1.64 ± 0.16	1.55 ± 0.26
Length/width ratio	2.29 ± 0.28	1.75 ± 0.15***	2.12 ± 0.35

Data are represented as mean ± standard deviation.

Welch’s *t*-test was used for statistical significance of differences between TauL1 and TauL2 or between subspecies *tauschii* and *stragulata* (** *P* < 0.01, *** *P* < 0.001).

PCA was performed for the grain shape-related traits of 37 TauL1 and TauL2 accessions, and PC1 and PC2 respectively explained 56.5 and 39.6% of the total variance (Table 3). A graph using the PC1 and PC2 values showed that the 37 accessions were grouped into TauL1, subspecies *tauschii* in TauL2, and subspecies *strangulata* in TauL2 (Fig 3C). TauL1 tended to have less than −0.5 of the PC1 values, and the subspecies *tauschii* and *strangulata* accessions in TauL2 generally had plus and minus values for PC2, respectively. As well as the spikelet shape-related traits, the grain shape traits were closely associated with intraspecific diversification of *Ae*. *tauschii*.

**Table 3:** Eigenvectors for the first and second principal components for the four grain shape traits.

Trait	PC1	PC2
Grain length	0.18	−0.75
Grain width	0.65	0.08
Grain height	0.58	−0.31
Length/width ratio	−0.45	−0.58

PC1 and PC2 explain 56.5 and 39.6% of the total variance, respectively.

### Evaluation of spikelet and grain shape-related traits in the mapping populations

Twelve and six spikelet and grain shape-related traits were respectively evaluated in the first two F_2_ populations and the third population of *Ae*. *tauschii*. Each trait showed widely distributed variation in the populations, and transgressive phenotypes were observed in each population (Tables 4 and 5), implying that several loci are contributed in the phenotypic variation. Parental accessions of TauL1 showed higher values for SL, NSp, SpD, GL, and LWr and lower values for NISp, SpL, SpW, EGL, GW, and GH than those of TauL2 in the three populations.

**Table 4:** Parental and F_2_ population means for eight spikelet-shape related traits in the three mapping populations.

	SL (cm)	NSp	NISp	SpD (cm^−1^)	SpL (mm)	SpW (mm)	EGL (mm)	EGW (mm)
KU-2078/PI499262								
KU-2078	9.62 ± 0.26**	12 ± 0***	1.33 ± 0.58*	1.25 ± 0.03*	9.56 ± 0.30***	4.57 ± 0.11***	5.93 ± 0.18	4.39 ± 0.06***
PI499262	11.43 ± 0.40	15.33 ± 0.58	0	1.34 ± 0.02	7.34 ± 0.07	2.60 ± 0.08	5.80 ± 0.04	2.63 ± 0.25
F_2_ population	107.65 ± 0.10	13.49 ± 0.99	1.00 ± 0.70	1.26 ± 0.10	8.87 ± 0.68	3.55 ± 0.32	6.27 ± 0.35	3.42 ± 0.29
Min-Max in F_2_	7.94–13.50	11.00–16.00	0.00–2.33	1.10–1.68	6.80–10.56	2.75–4.35	5.31–7.27	2.63–4.22
KU-2003/KU-2124								
KU-2003	11.61 ± 0.23	14.33 ± 0.58**	0.33 ± 0.58**	1.23 ± 0.02**	9.75 ± 0.28***	3.64 ± 0.17**	7.89 ± 0.41	3.32 ± 0.20 **
KU-2124	10.97 ± 0.40	12 ± 0	2 ± 0	1.10 ± 0.04	11.55 ± 0.09	4.38 ± 0.08	8.34 ± 0.05	4.06 ± 0.13
F_2_ population	11.65 ± 0.70	12.09 ± 1.12	1.03 ± 0.69	1.04 ± 0.09	10.15 ± 0.69	3.86 ± 0.22	7.98 ± 0.46	3.50 ± 0.25
Min-Max in F_2_	97.36–134.9	9.33–15	0–2.67	0.83–1.26	8.31–12.14	3.20–4.40	6.91–9.13	2.85–4.21
PI476874/IG47182								
PI476874	12.62 ± 0.52*	15.33 ± 1.15**	-	1.21 ± 0.04**	9.08 ± 0.46**	-	6.48 ± 0.44	3.77 ± 0.37
IG47182	11.31 ± 0.63	11.33 ± 0.58	-	1.00 ± 0.01	10.95 ± 0.33	-	6.60 ± 0.09	3.77 ± 0.15
F_2_ population	10.31 ± 0.99	10.32 ± 1.33	-	1.00 ± 0.09	10.13 ± 0.95	-	6.75 ± 0.50	3.62 ± 0.38
Min-Max in F_2_	7.6–12.7	7–13	-	0.83–1.27	7.90–12.40	-	5.90–8.40	3.05–6.45

Each trait value is represented as mean ± standard deviation.

Student’s *t*-test was used for statistical significance of parental differences (* *P* < 0.05, ** *P* < 0.01, *** *P* < 0.001).

**Table 5:** Parental and F_2_ population means for four grain-shape related traits in the two mapping populations.

	GL (mm)	GW (mm)	GH (mm)	LWr
KU-2078/PI499262				
KU-2078	4.68 ± 0.21	2.62 ± 0.11***	1.83 ± 0.16***	1.79 ± 0.09***
PI499262	4.81 ± 0.19	1.59 ± 0.15	1.10 ± 0.08	3.04 ± 0.31
F_2_ population	5.24 ± 0.30	2.36 ± 0.23	1.59 ± 0.18	2.24 ± 0.19
Min-Max in F_2_	3.70–5.79	1.79–2.78	0.87–1.90	1.74–2.81
KU-2003/KU-2124				
KU-2003	6.27 ± 0.12***	2.53 ± 0.12***	1.85 ± 0.12	2.48 ± 0.15***
KU-2124	5.93 ± 0.20	3.13 ± 0.17	1.90 ± 0.09	1.90 ± 0.12
F_2_ population	6.10 ± 0.29	2.76 ± 0.13	1.83 ± 0.11	2.21 ± 0.12
Min-Max in F_2_	5.08–6.67	2.33–3.04	1.51–2.08	1.93–2.52

Each trait value is represented as mean ± standard deviation.

Student’s *t*-test was used for statistical significance of parental differences (*** *P* < 0.001).

SL had higher positive correlation coefficients with SpL, SpW, EGL, and EGW in the first F_2_ population (KU-2078/PI499262) than in the other two populations (S2–S4 Tables). SpD was more negatively correlated with SL, SpL, SpW, EGL, and EGW in the first F_2_ population, whereas it correlated positively with NSp in the other populations. These results indicated that longer spikes and larger spikelets reduced spikelet density in this population, while the number of spikelets per spike contributed to low spikelet density in the others. Positive correlations between SpL, SpW, EGL, EGW, GL, and GW were commonly found in the three populations. However, strong or moderate positive correlations between GH and these six traits were observed only in the first F_2_ population, which might reflect the quadrate spikelets and spherical grains of subspecies *strangulata*. LWr was negatively correlated with SpW, EGW, and GW both in the first and second (KU-2003/KU-2124) F_2_ populations, but positively correlated with GL in the second F_2_ population.

### QTL analysis of spikelet morphological traits

In the first population, KU-2078/PI499262, 160 D-genome markers formed seven linkage groups, and total map length was 1250.7 cM with an average spacing of 7.85 cM between markers. The second mapping population, KU-2003/KU-2124, contained eight linkage groups, with two groups involved for chromosome 7D, and the total map length was 1455.4 cM with an average spacing of 13.2 cM between markers. In the third population, PI476874/IG47182, 158 markers were assigned to seven linkage groups, and the total map length was 1513.0 cM with an average spacing of 9.56 cM between markers.

**Fig 5:**
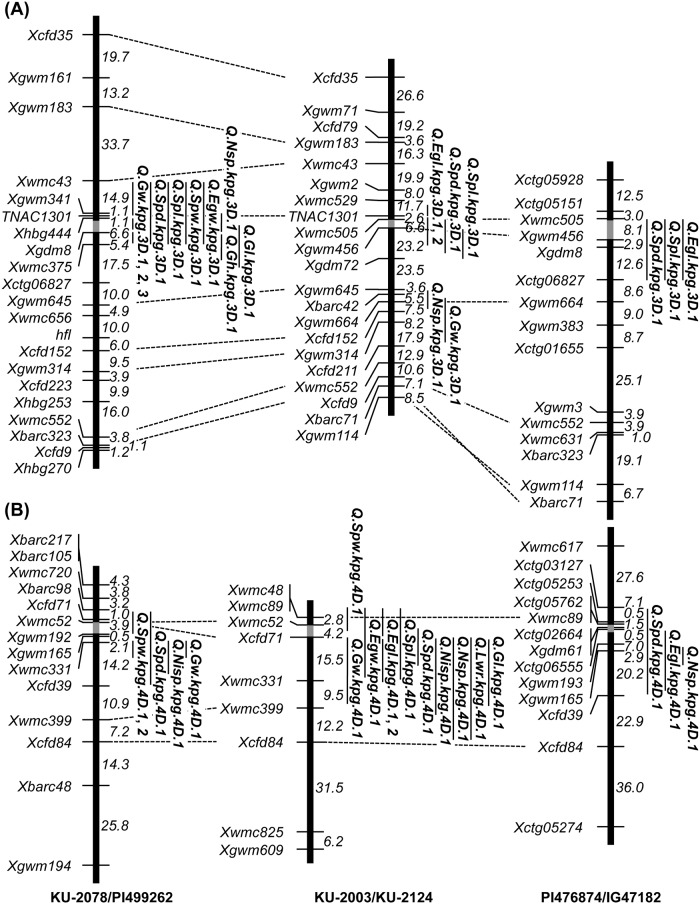
Comparison of the QTL positions on the chromosomes 3D (A) and 4D (B) linkage maps between the three *Ae*. *tauschii* populations. QTLs with LOD scores above the thresholds are indicated, and genetic distances are given in centimorgans. Gray boxes indicate putative centromeric regions.

**Fig 6:**
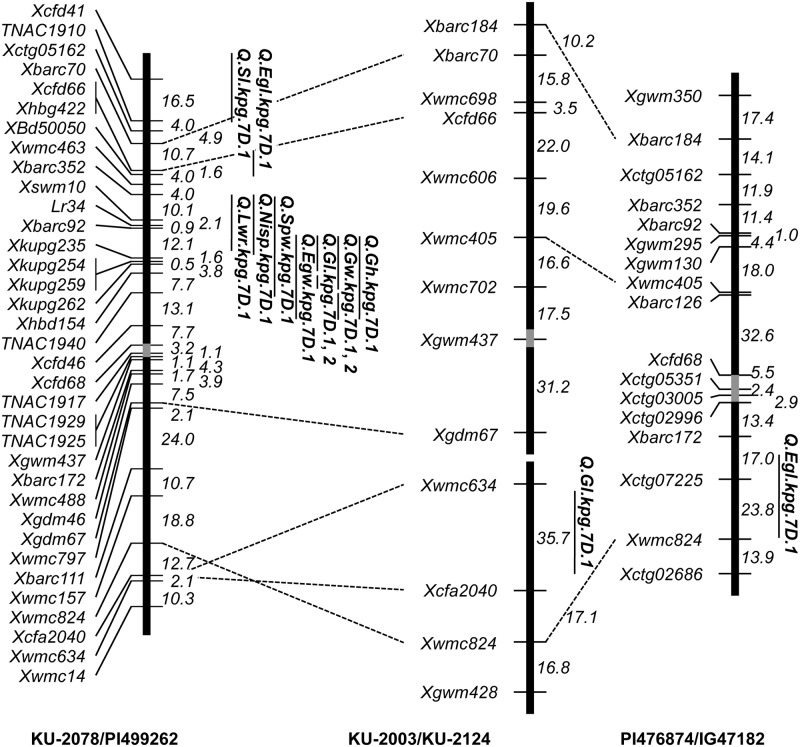
Comparison of the QTL positions on the chromosome 7D linkage maps between the three *Ae*. *tauschii* populations. QTLs with LOD scores above the thresholds are indicated, and genetic distances are given in centimorgans. Gray boxes indicate putative centromeric regions.

QTLs for all examined traits were detected using the three linkage maps. In the first F_2_ population, KU-2078/PI499262, 41 QTLs for the twelve examined traits were assigned to the seven *Ae*. *tauschii* chromosomes (Table 6). QTLs for NSp, SpD, SpL, SpW, EgW, GL, GW, and GH were detected on a similar proximal region of chromosome 3D, and QTLs for NISp, SpD, SpW, and GW overlapped on chromosome 4D (Fig 5). While, QTLs for four spikelet- and three grain-related traits were found within the interval between *Xbarc352* and *Xcfd46* on chromosome 7D (Fig 6).

**Table 6:** Summary of QTLs identified for spikelet- and grain-shape related traits in the KU-2078/PI499262 population.

Trait	Locus	Map location	LOD score	Contribution (%)	Additive effect
SL	*Q*.*Sl*.*kpg*.*1D*.*1*	*Xgwm458*–*Xhbg223*	15.49	41.5	−1.02
	*Q*.*Sl*.*kpg*.*5D*.*1*	*Xcfd266*–*Xgdm43*	5.52	12.1	0.53
	*Q*.*Sl*.*kpg*.*7D*.*1*	–*Xctg05162*	4.29	14.6	0.61
NSp	*Q*.*Nsp*.*kpg*.*1D*.*1*	*Xwmc432*–*Xcfd282*	9.67	27.9	−0.76
	*Q*.*Nsp*.*kpg*.*3D*.*1*	*Xgwm341*–*Xgdm8*	4.26	14.2	−0.60
	*Q*.*Nsp*.*kpg*.*5D*.*1*	*Xcfd266*–*Xgwm583*	3.95	14.7	0.59
	*Q*.*Nsp*.*kpg*.*6D*.*1*	*Xcfd132*–*Xcfd95*	9.22	34.3	−0.81
NISp	*Q*.*Nisp*.*kpg*.*1D*.*1*	*Xwmc432*–*Xgwm104*	4.29	12.2	−0.26
	*Q*.*Nisp*.*kpg*.*1D*.*2*	*Xgwm337*–*Xgwm458*	6.70	20.8	0.30
	*Q*.*Nisp*.*kpg*.*4D*.*1*	*Xgwm165*–*Xcfd39*	3.91	13.0	0.40
	*Q*.*Nisp*.*kpg*.*7D*.*1*	*Xbarc352*–*Xkupg235*	4.51	15.0	−0.40
SpD	*Q*.*Spd*.*kpg*.*1D*.*1*	*Xcfd48*–*Xwmc609*	4.55	11.7	0.03
	*Q*.*Spd*.*kpg*.*3D*.*1*	*Xwmc43*–*Xwmc375*	15.07	39.8	−0.06
	*Q*.*Spd*.*kpg*.*4D*.*1*	*Xbarc98*–*Xcfd39*	5.02	11.6	−0.03
SpL	*Q*.*Spl*.*kpg*.*1D*.*1*	*Xhbg223*–*Xgdm126*	5.16	13.6	−0.37
	*Q*.*Spl*.*kpg*.*3D*.*1*	*Xwmc43*–*Xwmc375*	9.32	25.9	0.55
	*Q*.*Spl*.*kpg*.*5D*.*1*	*Xgwm583*–*Xgdm43*	3.71	9.0	0.30
SpW	*Q*.*Spw*.*kpg*.*3D*.*1*	*Xwmc43*–*Xwmc375*	7.86	20.9	0.18
	*Q*.*Spw*.*kpg*.*4D*.*1*	*Xbarc98*–*Xgwm192*	3.80	9.3	0.15
	*Q*.*Spw*.*kpg*.*4D*.*2*	*Xgwm165*–*Xcfd39*	3.80	9.1	0.14
	*Q*.*Spw*.*kpg*.*7D*.*1*	*Xbarc352*–*Xcfd46*	11.22	30.0	0.14
EGL	*Q*.*Egl*.*kpg*.*1D*.*1*	*Xgwm337*–*Xbarc169*	9.24	24.0	−0.28
	*Q*.*Egl*.*kpg*.*5D*.*1*	*Xgdm138*–*Xgwm182*	9.43	26.6	0.36
	*Q*.*Egl*.*kpg*.*6D*.*1*	*Xcfd132*–*Xwmc753*	6.04	14.5	0.22
	*Q*.*Egl*.*kpg*.*7D*.*1*	*Xbarc70*–*Xwmc463*	4.37	8.6	0.09
EGW	*Q*.*Egw*.*kpg*.*1D*.*1*	*Xcfd282*–	4.33	10.9	−0.13
	*Q*.*Egw*.*kpg*.*3D*.*1*	*Xwmc43*–*Xwmc375*	7.54	16.8	0.17
	*Q*.*Egw*.*kpg*.*7D*.*1*	*Xbarc92*–*Xcfd46*	12.11	27.0	0.13
GL	*Q*.*Gl*.*kpg*.*3D*.*1*	*Xhbg444*–*Xctg06827*	5.22	18.3	0.19
	*Q*.*Gl*.*kpg*.*7D*.*1*	*Xbarc92*–*Xhbd154*	6.67	19.5	0.12
	*Q*.*Gl*.*kpg*.*7D*.*2*	*Xhbd154*–*TNAC1940*	4.85	15.7	0.04
GW	*Q*.*Gw*.*kpg*.*3D*.*1*	*Xwmc43*–*Xhbg444*	4.27	11.3	0.11
	*Q*.*Gw*.*kpg*.*3D*.*2*	*Xhbg444*–*Xgdm8*	3.98	10.8	0.10
	*Q*.*Gw*.*kpg*.*3D*.*3*	*Xgdm8*–*Xwmc375*	3.54	9.1	0.09
	*Q*.*Gw*.*kpg*.*4D*.*1*	*Xgwm165*–*Xcfd39*	3.63	8.6	0.09
	*Q*.*Gw*.*kpg*.*7D*.*1*	*Xbarc92*–*TNAC1940*	12.32	31.0	0.12
	*Q*.*Gw*.*kpg*.*7D*.*2*	*TNAC1940*–*Xcfd46*	3.88	10.2	0.04
GH	*Q*.*Gh*.*kpg*.*3D*.*1*	*Xgdm8*–*Xctg06827*	4.37	12.1	0.09
	*Q*.*Gh*.*kpg*.*7D*.*1*	*Xbarc92*–*Xcfd46*	15.91	43.7	0.12
LWr	*Q*.*Lwr*.*kpg*.*6D*.*1*	*Xcfd76*–*Xcfd38*	3.76	12.9	−0.10
	*Q*.*Lwr*.*kpg*.*7D*.*1*	*Xbarc352*–*Xkupg235*	8.54	32.4	−0.12

In the second population (KU-2003/KU-2124), 36 QTLs for these traits, except for GH, were assigned to the seven chromosomes (Table 7). QTLs for SpD, SpL, and EGL were found at a similar region of chromosome 3D, and six spikelet- and two grain-related QTLs were piled in the same region of the short arm of chromosome 4D (Fig 5).

**Table 7:** Summary of QTLs identified for spikelet- and grain-shape related traits in the KU-2003/KU-2124 and PI476874/IG47182 populations.

Trait	Locus	Map location	LOD score	Contribution (%)	Additive effect
KU-2003/KU-2124					
SL	*Q*.*Sl*.*kpg*.*2D*.*1*	*Xgwm30*–*Xgwm539*	4.19	14.5	−3.96
NSp	*Q*.*Nsp*.*kpg*.*3D*.*1*	*Xgwm645*–*Xcfd152*	4.22	8.2	0.42
	*Q*.*Nsp*.*kpg*.*4D*.*1*	*Xcfd71*–*Xcfd84*	13.47	32.5	0.98
NISp	*Q*.*Nisp*.*kpg*.*4D*.*1*	*Xcfd71*–*Xcfd84*	8.56	26.8	−0.49
	*Q*.*Nisp*.*kpg*.*6D*.*1*	*Xwmc435*–*Xbarc54*	4.33	10.6	−0.27
SpD	*Q*.*Spd*.*kpg*.*2D*.*1*	*PpdD1*–*Xgwm102*	5.37	12.8	0.27
	*Q*.*Spd*.*kpg*.*2D*.*2*	*Xgwm102*–*Xgwm515*	3.61	9.4	0.24
	*Q*.*Spd*.*kpg*.*3D*.*1*	*Xwmc505*–*Xgdm72*	3.80	17.2	0.24
	*Q*.*Spd*.*kpg*.*4D*.*1*	*Xwmc52*–*Xwmc331*	3.97	10.8	0.33
SpL	*Q*.*Spl*.*kpg*.*2D*.*1*	*Xgwm539*–*Xcfd168*	5.81	14.1	−0.37
	*Q*.*Spl*.*kpg*.*3D*.*1*	*Xgwm456*–*Xgdm72*	4.16	11.3	−0.33
	*Q*.*Spl*.*kpg*.*4D*.*1*	–*Xwmc331*	6.27	16.0	−0.58
	*Q*.*Spl*.*kpg*.*5D*.*1*	*Xcfd81*–*Xgwm213*	8.96	24.6	−0.49
SpW	*Q*.*Spw*.*kpg*.*2D*.*1*	–*Xwmc817*	4.54	14.3	0.03
	*Q*.*Spw*.*kpg*.*4D*.*1*	–*Xcfd71*	4.13	13.3	−0.12
	*Q*.*Spw*.*kpg*.*5D*.*1*	*Xcfd57*–*Xgdm153*	4.17	18.6	−0.15
EGL	*Q*.*Egl*.*kpg*.*1D*.*1*	*Xgwm106*–*Xcfd19*	5.11	12.1	0.26
	*Q*.*Egl*.*kpg*.*3D*.*1*	*Xwmc529*–*TNAC1301*	3.64	13.3	−0.19
	*Q*.*Egl*.*kpg*.*3D*.*2*	*TNAC1301*–*Xgdm72*	4.36	13.3	−0.23
	*Q*.*Egl*.*kpg*.*4D*.*1*	–*Xcfd71*	5.00	11.5	−0.26
	*Q*.*Egl*.*kpg*.*4D*.*2*	*Xcfd71*–*Xwmc331*	4.50	13.1	−0.27
EGW	*Q*.*Egw*.*kpg*.*2D*.*1*	*Xgwm539*–*Xcfd168*	5.63	13.7	−0.14
	*Q*.*Egw*.*kpg*.*4D*.*1*	–*Xwmc331*	7.33	18.6	−0.17
	*Q*.*Egw*.*kpg*.*5D*.*1*	*Xgdm153*–*Xwmc788*	6.31	18.2	−0.15
GL	*Q*.*Gl*.*kpg*.*2D*.*1*	*Xwmc245*–*Xwmc601*	4.25	10.8	0.11
	*Q*.*Gl*.*kpg*.*4D*.*1*	*Xcfd71*–*Xwmc399*	6.13	18.8	−0.17
	*Q*.*Gl*.*kpg*.*5D*.*1*	*Xgwm159*–*Xcfd266*	6.80	23.5	−0.20
	*Q*.*Gl*.*kpg*.*5D*.*2*	*Xwmc215*–*Xgdm63*	3.68	11.6	0.14
	*Q*.*Gl*.*kpg*.*6D*.*1*	*Xcfd188*–*Xbarc204*	3.68	10.4	0.06
	*Q*.*Gl*.*kpg*.*7D*.*1*	*Xwmc634*–*Xcfa2040*	5.06	26.4	0.22
GW	*Q*.*Gw*.*kpg*.*2D*.*1*	*Xgwm539*–*Xcfd168*	4.92	11.3	−0.07
	*Q*.*Gw*.*kpg*.*3D*.*1*	*Xbarc42*–*Xcfd211*	4.98	11.4	−0.06
	*Q*.*Gw*.*kpg*.*4D*.*1*	*Xcfd71*–*Xwmc399*	10.08	28.3	−0.12
LWr	*Q*.*Lwr*.*kpg*.*2D*.*1*	*Xwmc601*–*Xcfd233*	5.16	13.1	0.06
	*Q*.*Lwr*.*kpg*.*4D*.*1*	*Xcfd71*–*Xcfd84*	12.32	35.1	0.06
	*Q*.*Lwr*.*kpg*.*5D*.*1*	*Xwmc215*–*Xgdm63*	5.48	13.4	0.06
PI476874/IG47182					
NSp	*Q*.*Nsp*.*kpg*.*4D*.*1*	*Xgwm193*–*Xcfd39*	3.53	12.4	−0.74
SpD	*Q*.*Spd*.*kpg*.*3D*.*1*	*Xwmc505*–*Xctg06827*	8.97	24.4	−0.07
	*Q*.*Spd*.*kpg*.*4D*.*1*	*Xctg03127*–*Xcfd39*	8.05	22.7	−0.66
SpL	*Q*.*Spl*.*kpg*.*3D*.*1*	*Xgwm456*–*Xctg06827*	5.77	16.9	0.59
	*Q*.*Spl*.*kpg*.*5D*.*1*	*Xctg05398*–*Xgwm174*	5.12	14.8	0.51
EGL	*Q*.*Egl*.*kpg*.*3D*.*1*	*Xwmc505*–*Xctg06827*	6.25	17.5	0.29
	*Q*.*Egl*.*kpg*.*4D*.*1*	*Xctg06555*–*Xcfd39*	6.02	18.8	0.29
	*Q*.*Egl*.*kpg*.*7D*.*1*	*Xbarc172*–*Xwmc824*	5.45	15.6	−0.29

Eight QTLs for NSp, SpD, SpL, and EGL were assigned to four chromosomes in the third population, PI476874/IG47182 (Table 7), and three QTLs each overlapped on chromosomes 3D and 4D (Fig 5).

Comparison of the linkage maps and the QTL locations revealed that many spikelet- and grain-related QTLs were commonly detected on similar regions of chromosomes 3D and 4D in the three linkage maps (Fig 5). The spikelet- and grain-related QTLs on chromosome 7D were peculiar to the first population, KU-2078/PI499262, out of the three mapping populations (Fig 6). Several spikelet- and grain-related QTLs were found on the other four chromosomes; on chromosomes 1D, 5D and 6D in the first population and on chromosome 2D in the second population (Tables 6 and 7). Most of these QTLs covered different chromosomal regions in each mapping population and had no overlaps among the three populations. The SpL QTLs were found on chromosome 5D in all of the three mapping populations, whereas their locations on 5D were apparently distinct.

### Genotypic effects of the identified QTLs on spikelet morphology-related traits

**Fig 7:**
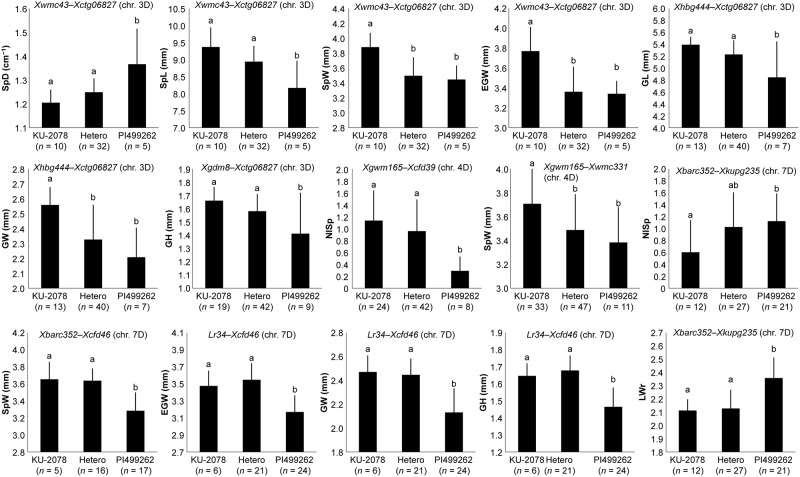
Genotypic effects of selected QTLs on spikelet-related traits in the KU-2078/PI499262 population. The genotypes of QTLs were inferred from genotyping data in the marker intervals shown above each graph. Means ± SD with the same letter were not significantly different (*P* > 0.05, Tukey-Kramer HSD test).

**Fig 8:**
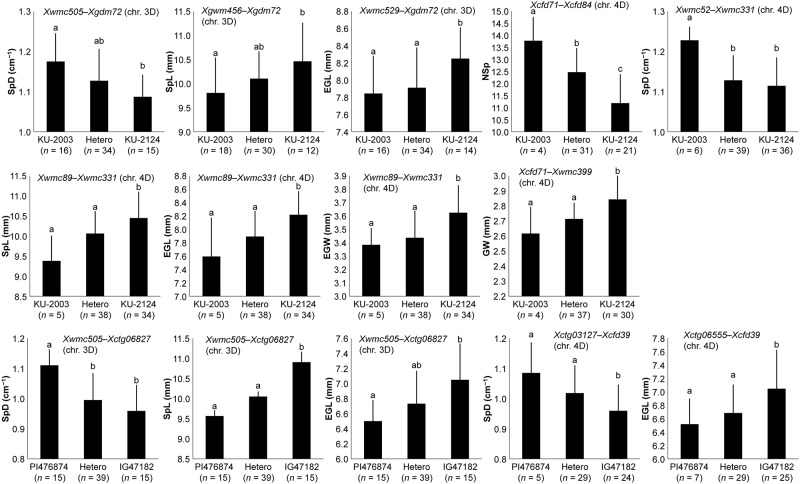
Genotypic effects of selected QTLs on spikelet-related traits in the KU-2003/KU-2124 and PI476874/IG47182 populations. The genotypes of QTLs were inferred from genotyping data in the marker intervals shown above each graph. Means ± SD with the same letter were not significantly different (*P* > 0.05, Tukey-Kramer HSD test).

The genotypes of the F2 individuals at these QTLs on chromosomes 3D, 4D, and 7D were deduced from genotyping data on the surrounding markers, and the genotypic effects of these QTLs were compared. Significant differences (Tukey-Kramer HSD test, *P* < 0.05) were observed between the F_2_ individuals carrying alleles from TauL1 and TauL2 at the 29 QTL regions (Figs 7 and 8). This observation was consistent with additive effects at each of the 29 QTLs, 27 of which coincided with the differences between the parents (Tables 6 and 7). No significant differences were observed between the F_2_ individuals with TauL1 type-homozygous, heterozygous, or TauL2 type-homozygous alleles at each of the following 5 QTLs; the 3D QTL for NSp and the 4D QTLs for SpD and GW in KU-2078/PI499262, the 4D QTL for LWr in KU-2003/KU-2124, and the 4D QTL for NSp in PI476874/IG47182. At the QTLs for GL, the 7D QTL in KU-2078/PI499262 and the 4D QTL in KU-2003/KU-2124, heterozygous individuals showed significantly higher values than TauL1 type -homozygous ones.

**Fig 9:**
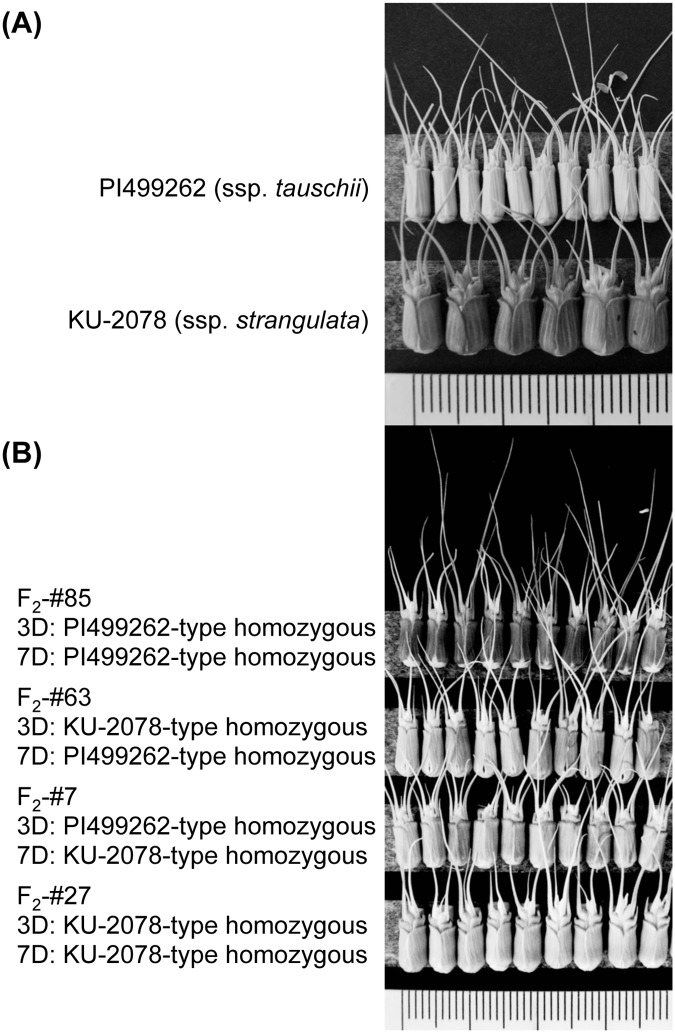
Phenotypic effect of the genotype combinations on spikelet morphology.

The parental accessions of the KU-2078/PI499262 population showed a distinct spikelet phenotype; SpW of the subspecies *strangulata* accession KU-2078 was much larger than that of the subspecies *tauschii* accession (Fig 9A). From the F_2_ individuals of KU-2078/PI499262, four F_2_ plants (#7, #27, #63 and #85) were selected based on genotyping data on the surrounding markers and their spikelets were compared (Fig 9B). An F_2_ plant (#27) with KU-2078-homozygous alleles for both the 3D and 7D QTLs exhibited larger SpW than the other three F_2_ plants. The spikelets of F_2_ plants (#7 and #63) with either the KU-2078-homozygous allele for the 3D or 7D QTL were wider than those with the PI499262-homozygous alleles (#85).

## Discussion

The wheat diploid D-genome progenitor, *Ae*. *tauschii*, carries large genetic variation in spikelet and grain morphology. Here, highly significant correlations among spikelet and grain shape-related traits were observed in the two mapping populations, which indicated a tight relationship between spikelet and grain shape (S2 and S3 Tables). In *Ae*. *tauschii*, the widths of spikelets, empty glumes, and grains determine sphericalness of grains. Similar observations have been reported in wheat and its relatives, and subspecies differentiation-related genes dramatically affect grain shape, accompanied by alteration of spikelet morphology in tetraploid and hexaploid wheat [27,42,43].

We detected a lot of QTLs on all seven chromosomes of *Ae*. *tauschii* for the analyzed traits, and assembled most to three chromosomal regions on chromosomes 3D, 4D and 7D. In the three mapping populations, the 3D and 4D QTL positions for the spikelet shape-related traits were consistent (Figs 5 and 6). Since the three populations were derived from interlineage crosses between TauL1 and TauL2, the 3D and 4D QTLs should contribute to intraspecific variation and lineage divergence of the spikelet morphology in *Ae*. *tauschii*. The TauL1 accessions provided longer spikes, higher spikelet density, and shorter and narrower spikelets than the TauL2 accessions (Fig 2), so intraspecific lineage divergence could be mainly caused by the genetic differences in the 3D and 4D QTL regions.

The three parental TauL1 accessions of the mapping populations were from TauL1b, which is considered to have spread eastward from the Transcaucasus and Middle East [12]. The TauL1a, TauL2 and TauL3 accessions are restricted to the Transcaucasus/Middle East region. Longitudinal clines for the spikelet morphology were observed in *Ae*. *tauschii*, and *Ae*. *tauschii* accessions in the eastern habitats tend to have the small-spikelet-size phenotype [10,11]. The TauL1b accessions showed different spike and spikelet shape-related traits from the TauL2 accessions (Figs 2 and 3), suggesting that the morphological clines, at least for spike and spikelet shape, could be due to the eastward expansion to Asia of TauL1b. Together with TauL1b, TauL1a also showed significant differences in spike and spikelet morphology compared with TauL2, and thus the morphological differences between the eastern and western accessions of *Ae*. *tauschii* might have arisen in the Transcaucasus/Middle East region before the TauL1b eastward expansion. The two QTL regions on chromosomes 3D and 4D should have the main contribution to the morphological divergence between the eastern and western accessions, and the effect of the 7D QTL region on the divergence could be additive.

Distinct spikelet and grain morphology of the two subspecies, *tauschii* and *strangulata*, is caused by genetic differences in the limited number of QTL regions (Table 6). The three QTLs on chromosomes 3D, 4D and 7D apparently mainly contribute to the morphological differentiation of the two subspecies (Figs 5 and 6). However, overlap of QTLs on chromosome 7D was found only in the intersubspecies population (subspecies *strangulata* and *tauschii*), not in intrasubspecies populations (subspecies *tauschii*), suggesting that these 7D QTLs are closely related to subspecies differentiation in *Ae*. *tauschii*. Within TauL2, therefore, the 7D QTL contributed to the morphological differentiation of the subspecies. The subspecies *strangulata* accessions are unevenly distributed in only the TauL2b and TauL2 admixture but not in TauL2a (S1 Table). The TauL2b accessions exhibited shorter spikes and spikelets and fewer spikelets per spike than the TauL2a accessions, whereas no clear difference for EGW was found between TauL2a and TauL2b (Fig 2). The limited effects of the 7D QTLs on the intersublineage divergence appear to be due to coexistence of the two subspecies within TauL2b, so the genetic difference in the 7D QTL region in TauL2 might have arisen within TauL2b.

Some spikelet- and grain-related QTLs were additionally found on chromosomes 1D, 2D, 5D and 6D (Tables 6 and 7), while the chromosomal regions for these QTLs showed few or no overlaps among the three mapping populations. These spikelet- and grain-related QTLs may reflect the morphological differences specific to the parental accessions and not be related to the lineage divergence and subspecies differentiation in *Ae*. *tauschii*. Therefore, other mapping populations derived from different parental combinations might allow us to detect additional QTLs for spikelet and grain shape in *Ae*. *tauschii*.

Genotypic differences in the 3D and 7D QTL regions additively affected the spikelet width (Fig 9), suggesting that the effects of the three QTLs on spikelet and grain morphology were additive. Addition of the genetic differences in the 7D QTL region to those of the 3D and 4D QTLs could more clearly have led to the subspecies differentiation in the spikelet and grain morphology. Due to the existence of morphological and genetic intermediates [16,44], the two subspecies were not formally described in a recent monograph [1]. In our previous reports [10,11], these subspecies can be separated when the sensu-stricto identification criteria are used. The Transcaucasian and Iranian accessions contain intermediates that should be assigned to subspecies *tauschii*. The intermediates are predicted to belong to TauL2 lacking the subspecies *strangulata*-type alleles at the 7D QTL regions, similarly to the TauL2 parental accessions of the second and third mapping populations, KU-2124 (TauL2a) and IG47182 (TauL2x). Thus, the genotype of the 7D QTL region could be a critical point to distinguish subspecies *strangulata* from subspecies *tauschii*. This assumption should be tested by construction of a fine map around the 7D QTL region and development of molecular markers closely linked to the 7D QTLs in further studies. Thus, QTL analysis for spikelet and grain shape of wild plant species provides useful information to elucidate the evolutionary processes leading to intraspecific differentiation.

Subspecies *strangulata* produces wider and more spherical grains than subspecies *tauschii*, and the three QTL regions of chromosomes 3D, 4D and 7D control the sphericalness of grains in subspecies *strangulata*. The birthplace of common wheat supposedly been restricted to a narrow distribution range within the western habitats of *Ae*. *tauschii* [45], and the D-genome donor accessions putatively belong to TauL2 or undiscovered populations [16,19,20]. Our previous study on the chromosomal region of *Iw2*, a repressor of glaucousness, suggests that the subspecies *strangulata* accessions do not represent the direct descendants of the ancestral populations that gave rise to common wheat [22]. Therefore, the 3D and 4D QTL regions could have been transmitted from the putatively ancestral TauL2 populations to common wheat, whereas the 7D QTLs might not have been integrated in the common wheat genome. Barley spike density is partly under the control of *dense spike-ar* (*dsp*.*ar*), assigned to the centromeric region of chromosome 7H [46]. The 7D QTL region we identified is apparently positioned on the short arm, distal to the centromere (Fig 6), indicating that the 7D QTLs might be not orthologous to barley *dsp*.*ar*.

Up to now, many QTLs for grain size and shape have been detected on various chromosomes of common wheat [30,47,48], whereas the 3D, 4D and 7D QTLs detected in the present study might be novel. No data have yet indicated that homoeologous loci of the 3D, 4D and 7D QTLs can be found on the A and B genomes of common wheat. To elucidate the homoeologous relationship to previously reported QTLs for grain size and shape, fine mapping of these QTLs will likely be required. Our previous study using synthetic wheat hexaploids derived from crosses between a tetraploid cultivar and some *Ae*. *tauschii* accessions showed the presence of several QTLs for grain size and shape on various D-genome chromosomes [43], whereas no QTL appears to correspond to these new 3D, 4D and 7D QTLs. The disappearance in the mapping populations of synthetic wheat might be due to suppression of the QTL effects by homoeologous loci of the A and B genomes in the hexaploid genetic background, although the reason is not clear. Therefore, simple introgression of the 7D QTL region through synthetic wheat might not be effective in significantly altering the grain shape of modern common wheat cultivars. For dramatic alteration of the grain shape, changes of the homoeologous alleles on the A and B genomes should be required along with the D-genome QTLs.

## Supporting information

S1 Table List of the *Ae*. *tauschii* accessions, lineage information and phenotypic data. (XLSX)

S2 Table Correlation coefficient (r) matrix for eight spikelet- and four grain-shape related traits in the KU-2078/PI499262 populations. Levels of significance are indicated by asterisks (* *P* < 0.05, ** *P* < 0.01, *** *P* < 0.001).
(PDF)

S3 Table Correlation coefficient (r) matrix for eight spikelet- and four grain-shape related traits in the KU-2003/KU-2124 populations. Levels of significance are indicated by asterisks (* *P* < 0.05, ** *P* < 0.01, *** *P* < 0.001).
(PDF)

S4 Table Correlation coefficient (r) matrix for five spikelet-shape related traits in the PI476874/IG47182 populations. Levels of significance are indicated by asterisks (* *P* < 0.05, ** *P* < 0.01, *** *P* < 0.001).
(PDF)
