# Supplementary material for: Quantitative trait locus analysis for spikelet shape-related traits in wild wheat progenitor Aegilops tauschii: Implications for intraspecific diversification and subspecies differentiation
Source: PLoS One. 2017 Mar 6;12(3):e0173210. doi: 10.1371/journal.pone.0173210 (PMC5338802; doi:10.1371/journal.pone.0173210)
Supplement: S2 Table — Levels of significance are indicated by asterisks (* P < 0.05, ** P < 0.01, *** P < 0.001). (PDF) [file pone.0173210.s002.pdf]

**S2 Table. Correlation coefficient (r) matrix for eight spikelet- and four grain-shape related traits in the KU-2078/PI499262 populations.**

| | NSp | NISp | SpD | SpL | SpW | EGL | EGW | GL | GW | GH | LWr |
|---|---|---|---|---|---|---|---|---|---|---|---|
| SL | 0.667*** | −0.114 | −0.647*** | 0.662*** | 0.455*** | 0.626*** | 0.415*** | 0.498*** | 0.375*** | 0.397*** | −0.091 |
| NSp | | −0.021 | 0.127 | 0.030 | 0.083 | 0.136 | 0.046 | −0.023 | 0.062 | 0.035 | −0.100 |
| NISp | | | 0.127 | −0.034 | 0.023 | −0.015 | −0.074 | −0.220* | −0.048 | −0.17 | −0.106 |
| SpD | | | | −0.847*** | −0.528*** | −0.694*** | −0.515*** | −0.718*** | −0.441*** | −0.541*** | 0.002 |
| SpL | | | | | 0.683*** | 0.731*** | 0.658*** | 0.678*** | 0.549*** | 0.559*** | −0.172 |
| SpW | | | | | | 0.51*** | 0.940*** | 0.528*** | 0.850*** | 0.721*** | −0.640*** |
| EGL | | | | | | | 0.534*** | 0.582*** | 0.426*** | 0.463*** | −0.099 |
| EGW | | | | | | | | 0.527*** | 0.853*** | 0.720*** | −0.646*** |
| GL | | | | | | | | | 0.566*** | 0.768*** | 0.025 |
| GW | | | | | | | | | | 0.803*** | −0.805*** |
| GH | | | | | | | | | | | −0.418*** |

Levels of significance are indicated by asterisks (* $P < 0.05$, ** $P < 0.01$, *** $P < 0.001$).
