# Supplementary material for: Quantitative trait locus analysis for spikelet shape-related traits in wild wheat progenitor Aegilops tauschii: Implications for intraspecific diversification and subspecies differentiation
Source: PLoS One. 2017 Mar 6;12(3):e0173210. doi: 10.1371/journal.pone.0173210 (PMC5338802; doi:10.1371/journal.pone.0173210)
Supplement: S3 Table — Levels of significance are indicated by asterisks (* P < 0.05, ** P < 0.01, *** P < 0.001). (PDF) [file pone.0173210.s003.pdf]

S3 Table. Correlation coefficient (r) matrix for eight spikelet- and four grain-shape related traits in the KU-2003/KU-2124 populations.

	NSp	NISp	SpD	SpL	SpW	EGL	EGW	GL	GW	GH	LWr
SL	0.4***	−0.148	−0.258**	0.311***	0.244**	0.381***	0.189*	0.206*	0.109	−0.028	0.094
NSp		−0.751***	0.781***	−0.312***	−0.091	−0.346***	−0.181	−0.073	−0.381***	0.019	0.275**
NISp			−0.692***	0.139	−0.051	0.255**	0.068	−0.058	0.278**	−0.026	−0.303***
SpD				−0.528***	−0.251**	−0.618***	−0.314***	−0.216*	−0.469***	0.043	0.221*
SpL					0.467***	0.616***	0.508***	0.408***	0.561***	−0.005	−0.141
SpW						0.522***	0.753***	0.274**	0.608***	0.306***	−0.301**
EGL							0.476***	0.39***	0.516***	0.184*	−0.109
EGW								0.287**	0.725***	0.129	−0.401***
GL									0.38***	0.369***	0.547***
GW										0.195*	−0.565***
GH											0.145

Levels of significance are indicated by asterisks (* $P < 0.05$, ** $P < 0.01$, *** $P < 0.001$).
