# Supplementary material for: Quantitative trait locus analysis for spikelet shape-related traits in wild wheat progenitor Aegilops tauschii: Implications for intraspecific diversification and subspecies differentiation
Source: PLoS One. 2017 Mar 6;12(3):e0173210. doi: 10.1371/journal.pone.0173210 (PMC5338802; doi:10.1371/journal.pone.0173210)
Supplement: S4 Table — Levels of significance are indicated by asterisks (* P < 0.05, ** P < 0.01, *** P < 0.001). (PDF) [file pone.0173210.s004.pdf]

S4 Table. Correlation coefficient (r) matrix for five spikelet-shape related traits in the PI476874/IG47182 populations.

	NSp	SpD	SpL	EGL	EGW
SL	0.708***	−0.043	0.398***	0.362***	0.268**
NSp		0.673***	−0.051	−0.097	0.119
SpD			−0.487***	−0.506***	−0.1
SpL				0.621***	0.237*
EGL					0.311**

Levels of significance are indicated by asterisks (* $P < 0.05$, ** $P < 0.01$, *** $P < 0.001$).
